# Sarcopenia in Patients With Spinal Metastasis: A Systematic Review and Meta-Analysis of Retrospective Cohort Studies

**DOI:** 10.3389/fonc.2022.864501

**Published:** 2022-04-05

**Authors:** Haifeng Tan, Xiaoyu Gao, Xiaoyu Li, Yunling Huang, Qi Cao, Teng Wan

**Affiliations:** ^1^ Hengyang Medical College, University of South China, Hengyang, China; ^2^ Department of Spine Surgery, The Second Affiliated Hospital, University of South China, Hengyang, China

**Keywords:** sarcopenia, spine, metastasis, meta, retrospective study

## Abstract

**Background:**

As a metastasis cancer that happens up to 70% of the cancer patients, spinal metastasis is drawing attention for its significant impairment to health. There exist several predictive models designed to estimate mortality in spinal metastasis patients but they are reported with limited accuracy. In recent years, some retrospective cohort studies have been carried out to associate sarcopenia with mortality in spinal metastasis.

**Introduction:**

As a risk factor leading to adverse events in many diseases, sarcopenia was considered to significantly impact on patients with spinal metastasis in mortality by some scientists. We aimed to look through the current evidence and use statistic measures to value the role of sarcopenia in spinal metastasis. In this study, we are going to perform a systematic review and meta-analysis of available retrospective cohort studies where sarcopenia is assessed for outcomes in spinal metastasis patients.

**Methods:**

On October 7, 2021, we performed a search in PubMed, Embase, and the Cochrane Library. We set no restrictions on language, date or areas. Results were expressed as hazard ratio (HR) or odds ratio (OR) with 95% CI by random effects model. Sensitivity analyses were performed to explore sources of heterogeneity and stability of results.

**Results:**

Of the 4,196 papers screened, 10 retrospective cohort studies were included, with a total of 1,674 patients. Results showed that sarcopenia was associated with higher overall mortality (OR, 1.60; 95% CI 1.35–1.90) and lower overall survival (HR, 2.08; 95% CI 1.55–2.80). The sensitivity analysis proved the stability of results in terms of publication years, region, time of diagnosis, sample size, female rate, measurement and follow up period.

**Conclusions:**

Sarcopenia is a robust indicator of mortality in spinal metastasis patients and it might be applied to decision-making tools to assess survival probability and adjust the extent of treatment, while a lack of higher level of evidence is existing.

**Systematic Review Registration:**

PROSPERO CRD42021283348.

## Introduction

Up to 70% of cancer patients develop secondary spinal metastasis, suffering from structural changes of the bone. With a progress in original cancer treatment, the metastasis is becoming more relevant (1). The surgery or immunotherapy effects of spinal metastasis are uncertain and patients may be selected for treatment without clear estimate of possible outcomes, such as survival rate and therapeutic options. Current predictive models designed to estimate mortality in patients with spinal metastasis are described with limited accuracy, though an improvement has been made in patients due to advances in multimodal therapy (2–5). Surgical decision-making tools like Tomita, Tokuhashi, Bauer, Van der Linden, Bollen, and Rades help doctors assess survival probability and adjust the extent of treatment, but ignore the significance of variables such as sarcopenia (2, 6).

As a skeletal muscle disorder affects muscle mass and function, sarcopenia is regarded as a risk factor that leads to adverse events in diseases (7–10). Sarcopenia has been shown by systematic reviews to negatively influence outcomes in digestive, cardiovascular, orthopedic diseases and tumor treatment in terms of survival rates, physical activity, length of hospital stay and other complications (11–17). Shachar et al. performed a meta-analysis confirming sarcopenia was risky on overall survival in patients with solid tumors (18). In recent years, many studies have been conducted to evaluate the prediction ability of sarcopenia on spinal metastasis, especially focused on mortality or survival (19).

The common measurement of sarcopenia is by computed tomography (CT) scans, but the selection of muscle varies in different studies. Psoas muscle size has been shown to predict perioperative outcomes and mortality after abdominal surgery (20). Total psoas muscle surface area (TPA) divided by vertebral body area (VBA) has been depicted to predict the likelihood of survival in metastatic spinal cord compression patients (21). We cannot find a clear definition of measurement for sarcopenia.

To clarify whether sarcopenia is predictive of survival in patients with spinal metastasis, we performed a systematic review of studies focusing on relationships between sarcopenia and outcomes in patients with cancer metastasis to the spine.

## Methods

The results were reported using the Preferred Reporting Items for Systematic Reviews and Meta-Analyses (PRISMA) for systematic reviews and meta-analyses (22) and the Meta-analysis of Observational Studies in Epidemiology (MOOSE) recommendations (23).

### Data Sources and Searches

We searched the PubMed, Embase, and the Cochrane Library using the terms Sarcopenia/Muscle Strength/Physical Fitness/Geriatric Assessment, Neoplasm Metastasis up to October 7, 2021. In addition, articles listed in the reference lists and related reviews were carefully selected identified. Only literature published in English were included (**Supplementary Material 1**).

### Study Selection

Two authors independently reviewed the title and abstract of each identified article and selected articles that might meet the criteria, and then read the full text of each selected literature to finish selection. Inclusion criteria were established *a priori*.

Population: Patients with spinal metastasis.
Comparator: sarcopenia patients versus non-sarcopenia patients
Outcome: mortality and survival
Study design: retrospective cohort study

Only original studies and conference abstract with available data were included.

### Data Extraction and Quality Assessment

Two authors independently extracted participant characteristics, namely, study design, region, diagnosed period, sample size, female%, original cancer type, measurements of sarcopenia, sarcopenia definition, outcomes, and follow-up period. Disagreement was resolved by discussion and consulting with the senior author (In some articles, there was no definition of sarcopenia but a divide of muscle mass into 3 tertile. Finally, we defined the 1st tertile as sarcopenia.) The quality score was derived by the Cochrane Collaboration’s tool and the Newcastle Ottawa Scale (24) (**Supplementary Material 2**), where selected items regarding the representativeness of the patients, ascertainment of exposure and outcomes, and adequacy of follow-up (25). We scored the quality ranging from 0 to 9 points for each study and defined a score of 8 or 9 as high-quality.

### Statistical Analysis

The primary outcomes analyzed were overall survival and overall mortality. Overall survival, defined as the time from surgery to death or the last follow-up, was calculated by HR. Pielkenrood’s study depicted 365-day mortality as HR, we took its reciprocal and defined it as overall survival. Overall mortality is defined as the time from surgery to death or the last follow-up or 1-year mortality.

We used Stata software (version 12.0) for data analysis. To meta-analyze the effect estimates (HRs) of overall survival, we applied random-effects models (the DerSimonianeLaird method), accounting for heterogeneity among studies (26). The risk estimates (HRs) were transformed into log HRs, along with their corresponding 95% CIs (27). To meta-analyze the effect estimates (ORs) of overall mortality, we converted reported ORs to log ORs and used a generalized inverse variance method with a random effects model combining data. Results are reported with both effect estimates and 95% confidence intervals (CIs). We used the I^2^ statistic to assess heterogeneity between studies, with I^2^ values >50% indicating significant heterogeneity (28). Begg’s funnel plot was used to detect publication bias in studies reporting overall survival, with a P-value <0.1 indicating a significant difference (29).

## Results

### Search Results

**Figure 1:**
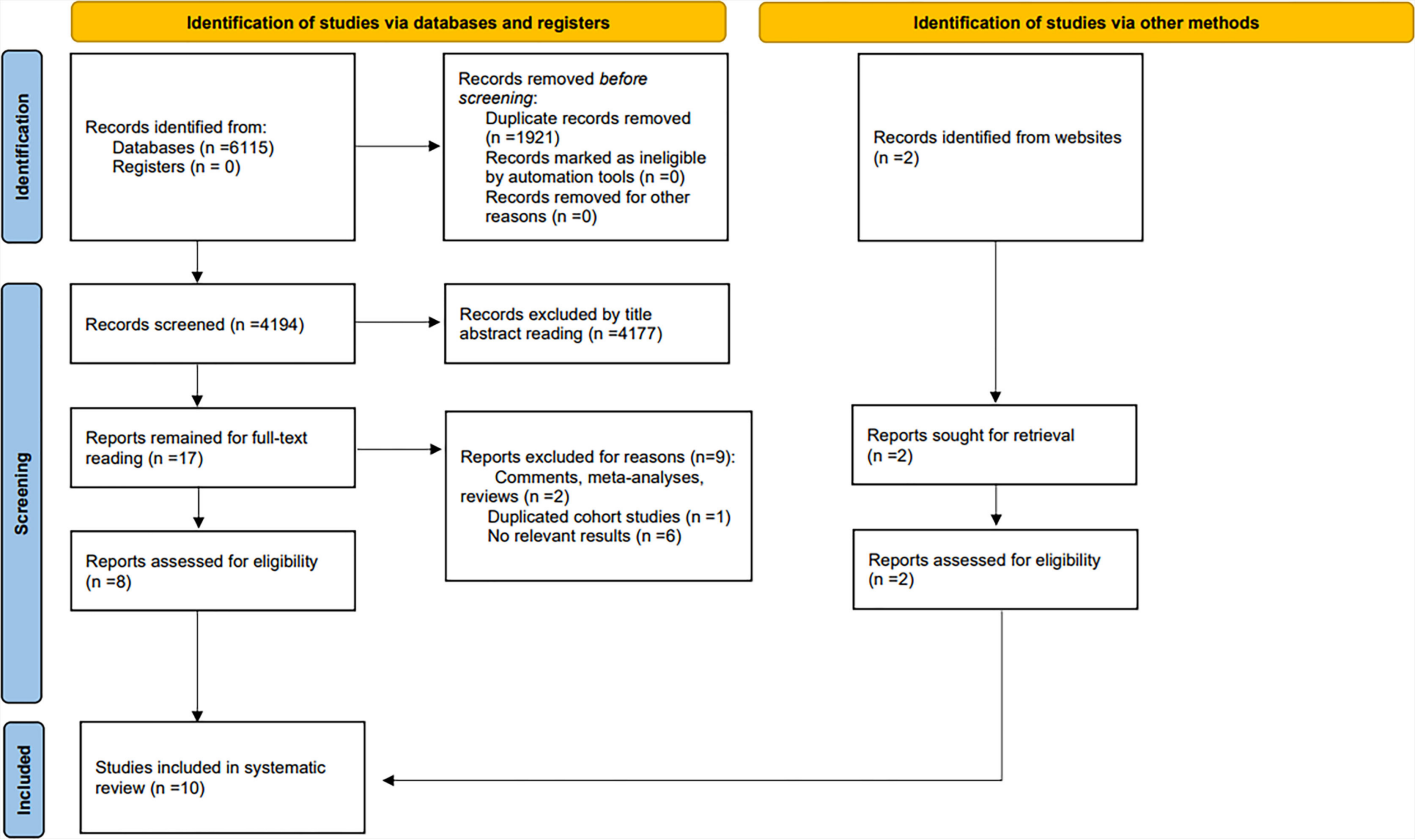
Flow diagram for the selection of studies.

We identified a total of 4,196 documents from the systematic literature search, of which 8 were evaluated for eligibility. In addition, a scan in the reference lists and related reviews was conducted to obtain 2 eligible studies. Finally, 10 eligible studies containing 1,674 patients were included. We excluded 2 comments, meta or review-type articles and 1 duplicate cohort study and 6 studies for which no relevant data were available. These studies were conducted in 3 countries on 3 continents: the USA, Netherlands, and Japan. The search and screening process is detailed in the PRISMA flowchart (**Figure 1**). Details of the included studies are shown in **Tables 1**, **2**.

**Table 1:** Characteristics of studies included in meta-analysis of sarcopenia for spinal metastasis.

First author	Year	Study design	Region	Diagnosedperiod	Sample size	Female%	Median age	Original cancer type
Massaad	2021	Retrospective cohort study	USA	2010 to 2019	88	26.1	62	Renal cell carcinoma
Zakaria1	2020	Retrospective cohort study	USA	1999 to 2017	271	42.1	57.4–61.3	Lung, prostate, kidney, liver, breast, hematopoietic, nasopharynx, skin, thyroid gastrointestinal
Zakaria2	2020	Retrospective cohort study	USA	2002 to 2012	417	51	65.3	Lung, breast, prostate, myeloma
Pielkenrood	2020	Retrospective cohort study	Netherlands	2013 to 2016	310	37	67	Lung, prostate, breast and other
Dohzono	2019	Retrospective cohort study	Japan	2009 to 2016	78	44	68.3	Gastrointestinal cancer
Zakaria3	2018	Retrospective cohort study	USA	2002 to 2012	92	NR	72.8	Prostate cancer
Zakaria4	2018	Retrospective cohort study	USA	2002 to 2012	118	100	63.8	Breast cancer
Zakaria5	2018	Retrospective cohort study	USA	2002 to 2012	46	43.7	63.2	Multiple myeloma
Zakaria6	2016	Retrospective cohort study	USA	2002 to 2012	168	46	64	Lung cancer
Gakhar	2015	Retrospective cohort study	USA	2009 to 2013	86	48.9	62–68	Breast, lymphoma, gastrointestinal, prostate, renal, lung and other

**Table 2:** Characteristics of studies included in meta-analysis of sarcopenia for spinal metastasis.

First author	Year	Measurements of sarcopenia	Sarcopenia definition	Treatment	Outcomes	Follow-up period
Massaad	2021	L3-SMI	Males: <43 cm^2^/m^2^ with BMI <25, <53 cm^2^/m^2^ with BMI >25 Females: <41 cm^2^/m^2^	Surgery	Overall mortality	1 to 104 months
Zakaria1	2020	PS	Male: <10.5 cm^2^ Female: <7.2 cm^2^	Surgery	Overall survival	NR
Zakaria2	2020	Average psoas/VBA	1st tertile	Radiation therapy, or with surgery	Overall survival	NR
Pielkenrood	2020	TPA/height^2^	Male:<52.4 cm^2^/m^2^ Female:<38.5 cm^2^/m^2^	Radiation therapy	Overall survival Overall mortality	2 to 5 years
Dohzono	2019	Paravertebral muscles	Less than median	Chemotherapy or surgery	Overall survival	1 to 8 years
Zakaria3	2018	Average psoas/VBA	1st tertile	Radiation therapy	Overall survival	NR
Zakaria4	2018	PS	1st tertile	Radiation therapy	Overall survival	600 days
Zakaria5	2018	Average psoas/VBA	1st tertile	Radiation therapy	Overall survival	5 years
Zakaria6	2016	Average psoas/VBA	1st tertile	Radiation therapy	Overall survival	5 years
Gakhar	2015	TPA/VBA	Lowest quartile	Surgery	Overall mortality	1 year

L3-SMI, L3 skeletal muscle index; PS, Psoas size; TPA, total psoas area; VBA, vertebral body area. BMI, body mass index; NR; not reported.

### Study Characteristics

Design of included studies: Retrospective cohort studies.

Original cancer type: Lung, prostate, kidney, breast, hematopoietic, gastrointestinal, nasopharynx, thyroid, liver, skin, myeloma, lymphoma.

Measurements of sarcopenia: One study used L3 skeletal muscle index (L3-SMI), two studies used psoas size (PS), four studies used average psoas/vertebral body area (VBA), one study used total muscle area, one study used paravertebral muscles, and one study used TPA/VBA. L3-SMI meant measuring the cross-section area of skeletal muscles (cm^2^) at L3 disc space divided by the square of the height of the patient (m^2^). The muscles included psoas, erector spinae, quadratus lumborum, transversus abdominis, external and internal oblique, and rectus abdominis muscles; PS meant measuring the size of psoas muscle at the L3/4-disc space or the L4 pedicle; Average psoas/VBA meant average psoas muscle size at the L4 vertebral level divided by the size of L4 vertebral body; Total muscle area meant total muscle size at L3 vertebral level which was the same to L3-SMI; Paravertebral muscles were measured by aggregating the cross-sectional area (mm^2^) at the L3 level; TPA/VBA meant total psoas muscle size at the L4 vertebral level divided by the size of L4 vertebral body.

Sarcopenia definition: For study used L3-SMI, sarcopenia was defined as L3-SMI <41 cm^2^/m^2^ in women, <43 cm^2^/m^2^ in men with BMI <25 kg/m^2^, and <53 cm^2^/m^2^ in men with BMI >25 kg/m^2^.Studies used PS defined sarcopenia as Men: <10.5 cm^2^, Women: <7.2 cm^2^ or 1st tertile. For the study that used paravertebral muscles sarcopenia was defined as the size less than median. Studies that used TPA/VBA defined sarcopenia as the lowest quartile. Other studies defined sarcopenia as the 1st tertile.

### Analysis of Outcome Measures

#### Overall Survival

**Figure 2:**
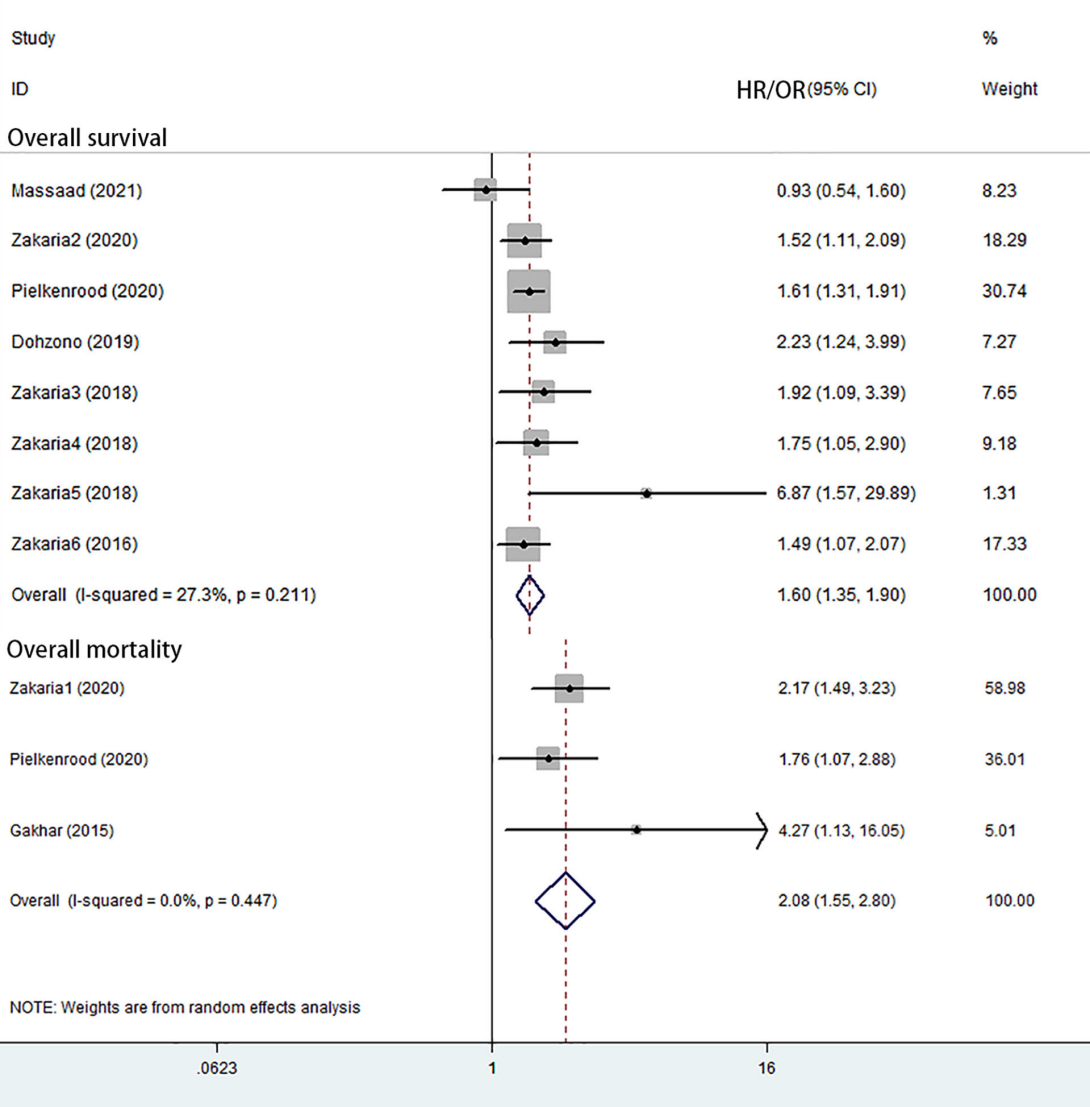
Forest plot of overall survival and overall mortality.

Eight studies reported overall survival (6, 30–36). Seven of these showed a significantly increased overall mortality related to sarcopenia. The random-effects meta-analysis showed that sarcopenia was associated with overall survival (HR = 1.60; 95% CI = 1.35–1.90; P-value <0.001) (**Figure 2** and **Table 3**).

**Table 3:** Meta analysis of outcomes.

Variables	HR/OR	95% Cl	p-Value for Association	I^2^ Value, %	p-Value for heterogeneity	Studies, n
Overall survival	1.60	1.35–1.90	<0.001	27.3	0.211	8
Overall mortality	2.08	1.55–2.80	<0.001	0	0.447	3

#### Overall Mortality

Three studies reported overall mortality (21, 31, 37). The random-effects meta-analysis showed that sarcopenia was associated with overall mortality (OR = 2.08; 95% CI = 1.55–2.80; P–value <0.001) (**Figure 2** and **Table 3**).

#### Risk of Bias and Quality Assessment

**Figure 3:**
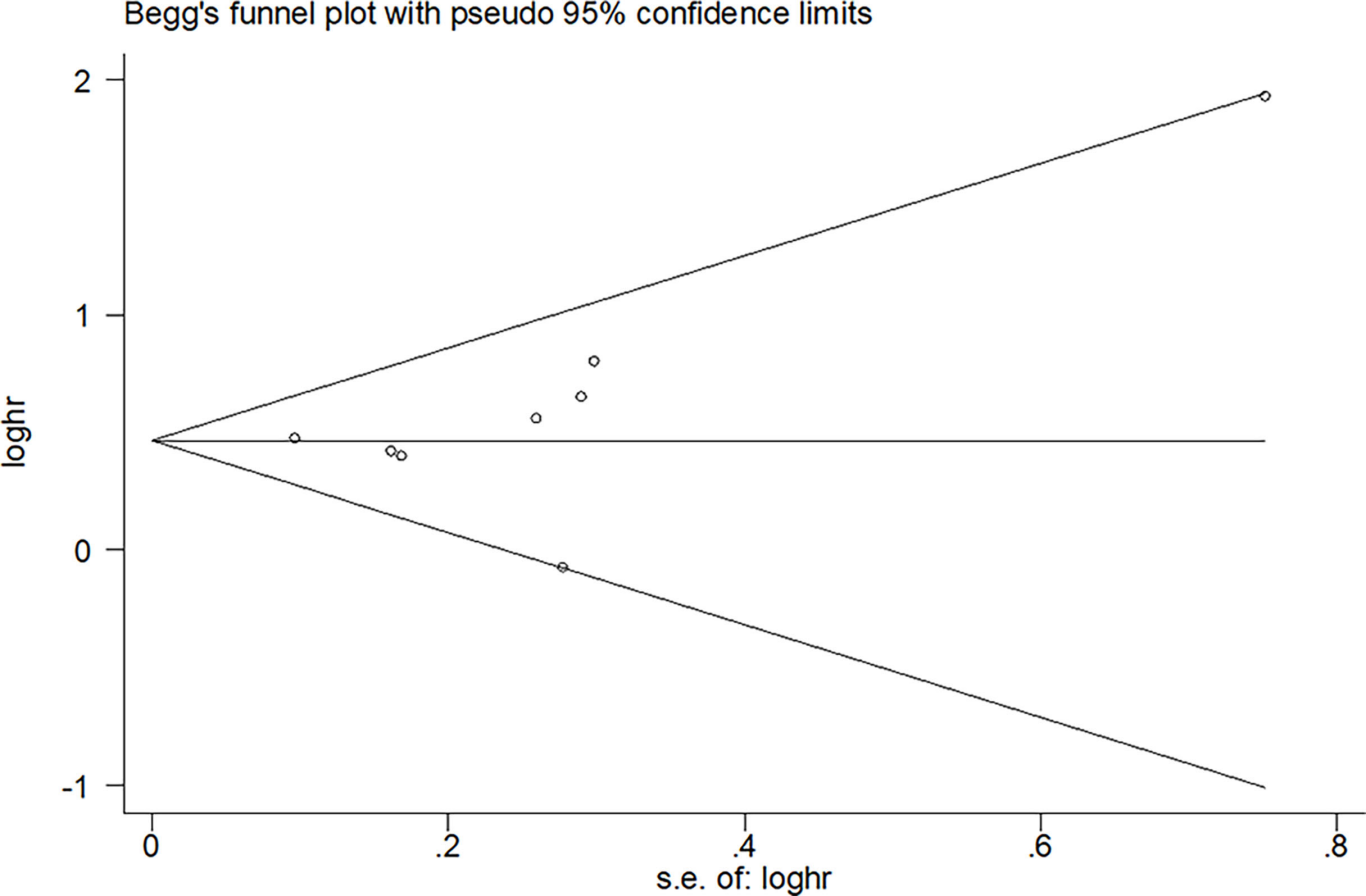
Publication bias of overall survival.

Both visual inspection funnel plots and Begg’s test suggested that no publication bias was found for overall survival, and Begg’s test was significant (Pr >|z| = 0.108) (**Figure 3**) (38). To assess the stability of the results, we performed sensitivity analyses. Criteria included: (1) publication in recent five years; (2) region in occident; (3) studies include diagnosis before 2010; (4) studies include diagnosis after 2015; (5) sample size >100; (6) female <50%; (7) exclude PS and L3-SMI; and (8) follow up longer than 2 years (**Supplementary Material 2**).

All results remained stable in the sensitivity analysis (**Supplementary Material 3**).

## Discussion

In this systematic review and meta-analysis, the results suggest that sarcopenia is likely to have an increased risk of mortality in patients with spinal metastasis. Our findings show that the pooled HR for survival among spinal metastasis patients with sarcopenia was 1.6 times higher than non-sarcopenia spinal metastasis patients. The ability to predict mortality was independent of publication years, region, diagnosed years, sample size, female rate, measurements, and follow up period. Since surgery for spinal metastasis may lead to higher mortality, neurological outcome, and pain, we came to a conclusion that sarcopenia may help in guiding treatment decision-making (39, 40).

In oncology surgery, sarcopenia has been applied to evaluate the risk of postoperative morbidity and survival of the patients. In the study by Sheetz, overall survival after esophagectomy for cancer was associated with core muscle size (P = 0.017) adjusted for age, gender, and stage (41). In hepatocellular carcinoma sarcopenia patients had lower survival (P = 0.012) and higher risk of low visceral fat area (p <0.001) (42). Otherwise, the similar association was proved in colorectal cancer and endometrial cancer (43, 44).

Sarcopenia and malnutrition often occur in the context of cancer and are also usually predictive of a poor prognosis (45). Therefore, detailed evaluation and regular monitoring of sarcopenia in the context of cancer is necessary. Nutritional care of cancer patients requires caution when treating sarcopenia, and the limited effectiveness of drugs and pharmacologic nutrients makes it necessary for cancer survivors to also exercise regularly to reduce the occurrence of sarcopenia (46). When sarcopenia occurs in the heart, heart failure and sarcopenia may reinforce each other. Heart failure may trigger sarcopenia due to hormonal changes, malnutrition and lack of physical activity, while sarcopenia may also promote the development of heart failure through pathological ergoreflex (47). Sarcopenia in heart failure is very common and is also associated with a poor prognosis, for which both nutritional and exercise therapies are important. Exercise, in particular, is the only treatment option for which there is sufficient clinical evidence (48). In addition, the use of drugs, ACE inhibitors and ARBs are both considered to have some muscoprotective effect, but the current clinical meta-analysis and basic studies on the role of this drug are still contradictory and further laboratory designs are needed to prove their effect (49). Sarcopenia occurs in the kidney when a negative nitrogen balance usually develops as chronic kidney disease progresses to its end stage (50). Therefore, sarcopenia due to uremia has more severe protein degradation on top of the primary sarcopenia and must restore appropriate exercise activity and adequate quality of life (51). Dietary interventions are considered to be a better way to ensure protein and energy intake in uremia to improve muscle mass reserve. However, it is important to note that according to epidemiological data, most of the good outcomes of reduced mortality associated with an oral nutritional high protein diet occur in individuals over 66 years of age (52). Current nutritional modalities for uremic sarcopenia generally include oral nutritional supplements, amino acids supplementation, intra-dialytic parenteral nutrition and enteral and total supplementation. Various nutritional modalities can help combat uremic rhabdomyosarcoma (53).

Due to the lack of an appropriate method and the limitation of content of included articles, we cannot carry on sensitivity analysis in terms of age, eventual hospitalization and oncological treatments. But we would like to discuss their impact on possible bias. The age of patients may correlate to mortality as sarcopenia happens more likely to old people and old patients are in commonly worse health condition (54). All the 10 articles are reported with a mean age over 60-y, and did not discuss young patients separately, so we have to be prudent when further studying this subject. All the 4,196 patients were in hospital, treated for spinal metastasis. We could not define who were considered as eventual hospitalization cases. The treatments for spinal metastasis in the 10 articles include surgery, radiotherapy and chemotherapy, which may differ from original cancer or life expectancy. When life expectancy is less than 3 months, a patient is not considered for surgery, as surgery takes time to recovery and is hard for him to justify (31). Sarcopenia seems to be predictive of mortality in 9 articles no matter which treatment is taken and the association between muscle mass and overall survival had been revealed independent of surgical procedure (30). Original cancer type, which was evaluated for sensitivity and proved the results stable, should be regarded attention to. The studies reported different original cancer types and some mixed several together. To clarify whether all original cancer types are sensitive to sarcopenia requires more specific studies.

Among the 10 included studies, only 1 study concluded that sarcopenia was not a risk factor to spinal metastasis which might be the result of strict inclusion criteria (6).This indicates that a unified criteria for selecting patients and operating method may reduce study bias (55, 56).

Though sarcopenia is widely studied by scientists, there is still a lack of consensus criteria and methods to investigate sarcopenia (57). The European Working Group on Sarcopenia in Older People advocates that the psoas is to be representative of sarcopenia (10). While other studies indicated that skeletal muscle in the level of L3 is associated well with whole body tissue mass in non-malignant populations (58, 59). In addition to muscle size, muscle strength and function might be factors to measure sarcopenia. These studies suggest that the use of different measurements for sarcopenia has a substantial conclusion on its effect.

Given the retrospective nature of these studies, we were unable to account for unintended bias and the heterogeneity of complications. The region was a limitation of our studies as most of the studies were carried in occident, a more convincing conclusion could be reached with more statistic from Asia, Africa, Latin America, and Oceania.

Given its consequences, sarcopenia might be applied to decision-making tools to assess survival probability and adjust extent of treatment, but there is not enough evidence to deem it as an independent predictor. Thus, sarcopenia should be considered in a multidisciplinary way and evaluated in complexity. Additionally, sarcopenia can be regarded as a vital health problem, and an effort to prevent and treat sarcopenia is requisite.

## Conclusions

In this article, we performed a systematic evaluation and meta-analysis of sarcopenia in spinal metastasis patients. The results suggest that sarcopenia might be an indicator of mortality in spinal metastasis patients. Sensitivity analysis on some baseline factors suggests that this relation is stable. However, there is still a need to conduct larger prospective cohort studies to confirm the conclusion.

## Data Availability Statement

The original contributions presented in the study are included in the article/**Supplementary Material**. Further inquiries can be directed to the corresponding authors.

## Author Contributions

HT and TW conceived and designed the experiments. HT and TW performed the experiments. HT and TW contributed material/analysis tools. HT, XG, and XL wrote the manuscript. TW, and YH performed reference collection and data management. HT performed statistical analyses. QC and TW critically revised and edited successive drafts of the manuscript. All authors listed have made a substantial, direct, and intellectual contribution to the work and approved it for publication.

## Conflict of Interest

The authors declare that the research was conducted in the absence of any commercial or financial relationships that could be construed as a potential conflict of interest.

## Publisher’s Note

All claims expressed in this article are solely those of the authors and do not necessarily represent those of their affiliated organizations, or those of the publisher, the editors and the reviewers. Any product that may be evaluated in this article, or claim that may be made by its manufacturer, is not guaranteed or endorsed by the publisher.
