# Supplementary material for: Sarcopenia in Patients With Spinal Metastasis: A Systematic Review and Meta-Analysis of Retrospective Cohort Studies
Source: Front Oncol. 2022 Apr 5;12:864501. doi: 10.3389/fonc.2022.864501 (PMC9037148; doi:10.3389/fonc.2022.864501)
Supplement: Supplementary file 1 [file DataSheet_1.docx]

**Supplementary Material 1**

**Text S1 Search strategy**

**Database: Pubmed from inception to Present> (Search date: October 7, 2021)**

**Search Strategy:**

--------------------------------------------------------------------------------

***Sarcopenia terms:***

1. "Sarcopenia"[Mesh]
2. "Muscle Strength"[Mesh]
3. "Physical Fitness"[Mesh]
4. "Geriatric Assessment"[Mesh]
5. (Morphometrics or Physical performance or Frail* or Geriatric Assessment or Physical function or Muscle strength or Muscle function or Hand grip strength or Gait speed or Walking speed or Fitness or Physical fitness or Body composition or Sarcopeni* or Cachexia or Skeletal muscle or Muscle mass or Skeletal muscle) [Title/Abstract]
6. 1-5/or

***Spinal metastases terms:***

1. "Neoplasm Metastasis"[Mesh]
2. (Metastatic or Neoplasm Metastases or Metastas* or spinal metastasis or Spinal Metastases or Metastases, Spinal or Metastasis, Spinal or Metastatic Spinal Cord Compression or Spinal Epidural Metastases or Vertebral Metastases or Vertebral Metastasis or Metastatic Spinal Tumors) [Title/Abstract]
3. 7-8/or

***Final search results: Combining Sarcopenia and Spinal metastases:***

1. 6 and 9 (2964)

**Text S2 Search strategy**

**Database: EMBASE (Search date: October 7, 2021)**

**Search Strategy:**

--------------------------------------------------------------------------------

***Sarcopenia terms:***

1. 'sarcopenia'/exp
2. 'muscle strength'/exp
3. 'fitness'/exp
4. 'geriatric assessment'/exp
5. (morphometrics:ab,ti OR 'physical performance':ab,ti OR frail*:ab,ti OR 'geriatric assessment':ab,ti OR 'physical function':ab,ti OR 'muscle strength':ab,ti OR 'muscle function':ab,ti OR 'hand grip strength':ab,ti OR 'gait speed':ab,ti OR 'walking speed':ab,ti OR fitness:ab,ti OR 'physical fitness':ab,ti OR 'body composition':ab,ti OR sarcopeni*:ab,ti OR cachexia:ab,ti OR 'muscle mass':ab,ti OR 'skeletal muscle':ab,ti) AND [embase]/lim
6. 1-5/or

***Spinal metastases terms:***

1. 'spine metastasis'/exp
2. (metastatic:ab,ti OR 'neoplasm metastases':ab,ti OR metastas*:ab,ti OR 'spinal metastasis':ab,ti OR 'spinal metastases':ab,ti OR 'metastases spinal':ab,ti OR 'metastasis spinal':ab,ti OR 'metastatic spinal cord compression':ab,ti OR 'spinal epidural metastases':ab,ti OR 'vertebral metastases':ab,ti OR 'vertebral metastasis':ab,ti OR 'metastatic spinal tumors':ab,ti) AND [embase]/lim
3. 7-8/or

***Final search results: Combining Sarcopenia and Spinal metastases:***

1. 6 and 9 (1237)

**Text S3 Search strategy**

**Database: Cochrane Library from inception to Present> (Search date: May 9, 2021)**

**Search Strategy:**

--------------------------------------------------------------------------------

***Sarcopenia terms:***

#1 MeSH descriptor: [Muscle Strength] explode all trees 6230

#2 MeSH descriptor: [Physical Fitness] explode all trees 3563

#3 MeSH descriptor: [Geriatric Assessment] explode all trees 1525

#4 MeSH descriptor: [Sarcopenia] explode all trees 544

#5 (Morphometrics or Physical performance or Frail* or Geriatric Assessment or Physical function or Muscle strength or Muscle function or Hand grip strength or Gait speed or Walking speed or Fitness or Physical fitness or Body composition or Sarcopeni* or Cachexia or Skeletal muscle or Muscle mass or Skeletal muscle):ti,ab,kw (Word variations have been searched) 144618

#6 #1 or #2 or #3 or #4 or #5 144865

***Spinal metastases terms:***

#7 MeSH descriptor: [Neoplasm Metastasis] explode all trees 5324

#8 (Metastatic or Neoplasm Metastases or Metastas* or spinal metastasis or Spinal Metastases or Metastases, Spinal or Metastasis, Spinal or Metastatic Spinal Cord Compression or Spinal Epidural Metastases or Vertebral Metastases or Vertebral Metastasis or Metastatic Spinal Tumors):ti,ab,kw (Word variations have been searched) 45102

#9 #7 or #8 45237

***Final search results: Combining Sarcopenia and Spinal metastases:***

#10 #6 and #9 1914
