# Supplementary material for: Sarcopenia in Patients With Spinal Metastasis: A Systematic Review and Meta-Analysis of Retrospective Cohort Studies
Source: Front Oncol. 2022 Apr 5;12:864501. doi: 10.3389/fonc.2022.864501 (PMC9037148; doi:10.3389/fonc.2022.864501)
Supplement: Supplementary file 2 [file DataSheet_2.docx]

**Supplementary Material 2. Methodological quality score of the included studies based on the Newcastle–Ottawa scale (NOS) tool.**

| **Author** | **Year** | **Study Design** | **Selection** | | | | **Comparability** | **Exposure/Outcome** | | | **Total Score** | **Risk of Bias** |
| --- | --- | --- | --- | --- | --- | --- | --- | --- | --- | --- | --- | --- |
|  |  |  | **Representative­ness of cohort *** | **Selection of control cohort *** | **Ascertainment of exposure *** | **Outcome not present at start *** | **Comparability of cohorts **** | **Assessment of outcome *** | **Length of follow-up *** | **Adequacy of follow-up *** | **Total score** |  |
| Massaad | 2021 | Retrospective cohort study |  | * | * | * | ** | * | * | * | 8 | low |
| Zakaria1 | 2020 | Retrospective cohort study |  | * | * | * | ** | * | * | * | 8 | low |
| Zakaria2 | 2020 | Retrospective cohort study |  | * | * | * | ** | * |  |  | 6 | high |
| Pielkenrood | 2020 | Retrospective cohort study |  | * | * | * | ** | * | * | * | 8 | low |
| Dohzono | 2019 | Retrospective cohort study |  | * | * | * | ** | * | * | * | 8 | low |
| Zakaria3 | 2018 | Retrospective cohort study |  | * | * | * | ** | * |  |  | 6 | high |
| Zakaria4 | 2018 | Retrospective cohort study |  | * | * | * | ** | * | * | * | 8 | low |
| Zakaria5 | 2018 | Retrospective cohort study |  | * | * | * | ** | * | * | * | 8 | low |
| Zakaria6 | 2016 | Retrospective cohort study |  | * | * | * | ** | * | * | * | 8 | low |
| Gakhar | 2015 | Retrospective cohort study |  | * | * | * | ** | * | * | * | 8 | low |
