# Supplementary material for: Sarcopenia in Patients With Spinal Metastasis: A Systematic Review and Meta-Analysis of Retrospective Cohort Studies
Source: Front Oncol. 2022 Apr 5;12:864501. doi: 10.3389/fonc.2022.864501 (PMC9037148; doi:10.3389/fonc.2022.864501)
Supplement: Supplementary file 3 [file DataSheet_3.docx]

**Supplementary Material 3. Sensitivity analyses.**

| Variables | HR/OR | 95%CI | p Value for Association | I^2^ Value, % | p Value for heterogeneity | Studies, n |
| --- | --- | --- | --- | --- | --- | --- |
| **Overall survival** | | | | | | |
| Recent five years | 1.64 | 1.33-2.02 | <0.001 | 36.5 | 0.150 | 7 |
| Occident | 1.56 | 1.31-1.86 | <0.001 | 27.6 | 0.218 | 7 |
| Including diagnosis before 2010 | 1.70 | 1.39-2.07 | <0.001 | 9.8 | 0.353 | 6 |
| Including diagnosis after 2015 | 1.51 | 1.02-2.23 | 0.040 | 60.2 | 0.081 | 3 |
| Sample size >100  Female<50%  Exclude PS and L3-SMI  Follow up longer than 2 years | 1.58  1.59  1.65  1.59 | 1.37-1.82  1.18-2.14  1.41-1.92  1.31-1.93 | <0.001  0.002  <0.001  <0.001 | 0  55.4  10.3  36.8 | 0.946  0.062  0.350  0.148 | 4  5  6  7 |
| **Overall mortality** | | | | | | |
| Recent five years | 2.00 | 1.48-2.72 | <0.001 | 0 | 0.514 | 2 |
| Including diagnosis before 2010  Including diagnosis after 2015  Sample size >100  Exclude PS and L3-SMI  Follow up longer than 2 years | 2.29  2.00  2.00  2.19  2.00 | 1.58-3.32  1.48-2.72  1.48-2.72  1.04-4.65  1.48-2.72 | <0.001  <0.001  <0.001  0.040  <0.001 | 0  0  0  33.5  0 | 0.337  0.514  0.514  0.220  0.514 | 2  2  2  2  2 |

Abbreviations: L3-SMI, L3 skeletal muscle index; PS, Psoas size;
